# Acknowledgement to reviewers

**DOI:** 10.1186/s41021-016-0048-6

**Published:** 2016-06-22

**Authors:** Takashi Yagi

**Affiliations:** Laboratory of Molecular and Cellular Genetics, Department of Biology, Graduate School of Science, Osaka Prefecture University, Osaka, Japan

## Abstract

The editors of *Genes and Environment* would like to thank all our reviewers who have contributed to the journal in Volume XXXVII (2015).

**Mie Akanuma**

Japan

**Yasunobu Aoki**

Japan

**Michael Fenech**

Australia

**Kazuo Fujikawa**

Japan

**Chie Furihata**

Japan

**Atsushi Hakura**

Japan

**Shuichi Hamada**

Japan

**Tsuneo Hashizume**

Japan

**Yuko Ibuki**

Japan

**Hiroshi Ide**

Japan

**Youhei Inaba**

Japan

**Keiko Inami**

Japan

**Satoru Itoh**

Japan

**Nishad Jayasundara**

United States of America

**Hiroyuki Kamiya**

Japan

**Kazuaki Kawai**

Japan

**Makoto Kobayashi**

Japan

**Seiji Kodama**

Japan

**Isao Kuraoka**

Japan

**Yingsong Lin**

Japan

**Yuji Masuda**

Japan

**Kenichi Masumura**

Japan

**Tomonari Matsuda**

Japan

**Kyomu Matsumoto**

Japan

**Nan Mei**

United States of America

**Takeshi Morita**

Japan

**Yoshimichi Nakatsu**

Japan

**Takehiko Nohmi**

Japan

**Shinji Oikawa**

Japan

**Juraci Alves de Oliveira**

Brazil

**Angela Risch**

Germany

**Kazuo Sakai**

Japan

**Megumi Sasatani**

Japan

**Kazuhiro Shiizaki**

Japan

**Yoshiya Shimada**

Japan

**Kazunori Shiraishi**

Japan

**Haruhiko Sugimura**

Japan

**Shizuyo Sutou**

Japan

**Hitoshi Suzuki**

Japan

**Takayoshi Suzuki**

Japan

**Akira Toriba**

Japan

**Arikuni Uchimura**

Japan

**Keiji Wakabayashi**

Japan

**Akihiro Wakata**

Japan

**Masahiko Watanabe**

Japan

**Tetsushi Watanabe**

Japan

**J Weller**

Israel

**Masami Yamada**

Japan

**Ayumi Yamamoto**

Japan

**Yuichiro Yokota**

Japan

**Huiping Zhang**

United States of America

